# Amplification, Resistance, and Kinetics of the Jaw Stretching Device (ARK-JSD): analysis of the force variation and implications for trismus therapy

**DOI:** 10.1007/s10006-024-01218-1

**Published:** 2024-02-06

**Authors:** Emma Charters, Kai Cheng, Masako Dunn, Aaron Luo, Y. M. Aung, Will Lewin, Jonathan R. Clark

**Affiliations:** 1 Department of Head and Neck Surgery, Chris O’Brien Lifehouse, 119-143 Missenden Road, Camperdown, Sydney, NSW 2050 Australia; 2 Faculty of Medicine and Health, Sydney School of Health Sciences, The University of Sydney, Camperdown, Sydney, NSW 2006 Australia; 3 Sydney Local Health District, Royal Prince Alfred Institute of Academic Surgery, Camperdown, Sydney, NSW 2050 Australia; 4 Sarcoma and Surgical Research Centre, Chris O’Brien Lifehouse, Camperdown, Sydney, NSW 2050 Australia; 5 Biomedical Innovation, Chris O’Brien Lifehouse, Camperdown, Sydney, NSW 2050 Australia; 6 Faculty of Medicine and Health, Sydney Medical School, The University of Sydney, Anderson Stuart Building, Camperdown, NSW 2006 Australia

**Keywords:** Trismus, Head and neck cancer, Oral cancer, Medical devices

## Abstract

**Purpose:**

Jaw-stretching devices, including the Amplification, Resistance, and Kinetics of the Jaw (ARK-JSD), are an effective option for treating trismus after head and neck cancer (HNC) treatment. The force, however, that is applied to the patient’s jaw is unknown.

**Methods:**

Ten ARK-JSD devices were constructed for each of the levels of resistance (total of 30 samples). Each sample was tested using a Universal Testing Machine (UTM).

**Results:**

The easy, medium, and hard ARK-JSD had a mean maximum force of 12.3, 21.0, and 32.7 Newtons (N) at a mean interincisal distance (IID) of 8.0 mm, 13.0 mm, and 16.0 mm, respectively. The force varied by 6.9 N for the easy and 24.1 N for the hard ARK-JSD. Fatigue analysis demonstrated up to 5.5 N loss of force over 10 weeks.

**Conclusion:**

The ARK-JSD is a low-cost trismus device that can force between 12.3 and 32.7 N. The variation in resistance may impact efficacy. Understanding this variation will assist clinicians and patients using the ARK-JSD for trismus therapy.

## Introduction

Trismus (restricted mouth opening) is a common problem after head and neck cancer treatment, particularly when multimodality therapy (surgery and/or chemoradiotherapy) is required for advanced cancers [1]. Trismus impairs access to the mouth for self-administered oral hygiene and professional dental care, which accelerates dental caries and loss of dentition [2]. It also can make eating in public more challenging, placing cancer patients with xerostomia and dysphagia at risk of malnutrition [3]. Together, these side effects lead to anxiety, impaired socialisation and even financial hardship through loss of employment [4–7]. Whilst there is evidence showing that jaw-stretching devices are effective in the treatment of trismus, commercial devices are often under-utilised because of their high cost [8]. The efficacy and safety of such devices may also be compromised because the force applied to the jaw is unknown and unregulated. This means that the patient determines what force is appropriate, and whilst therapy is often supervised by trained professionals, there is no way to know whether the force applied is sufficient to improve mouth opening or excessive in patients with compromised dentition or bone integrity.

Amplification, Resistance, and Kinetics of the Jaw (ARK-J) is a trismus training course which trains clinicians in trismus assessment and therapy. It also teaches clinicians how to make the homemade ARK-J Stretching Device (ARK-JSD) as a replacement for the common practice of using stacked tongue depressors for trismus therapy. The ARK-JSD’s key features are low cost, portability, and three levels of resistance (easy/medium/hard). These features allow patients who are unable to afford commercial devices to access a trismus therapy device which can both passively and actively exercise the jaw. The three different ARK-JSD construction options allow patients to progress by increasing the mechanical load to meet their rehabilitation goals. Whilst the ARK-J programme adheres to the exercise physiology principle of load progression [9], the specific load for each level of resistance has not been published. Furthermore, as the ARK-JSD is constructed by the clinician, it is unknown whether there is substantial variation in the force generated between each individual device for a given level of resistance and whether this force changes with time or use. This study aimed to define the mean load and sample variation of individually constructed ARK-JSDs at each level of resistance.

## Materials and methods

Ethical approval was not required for this non-biological in vitro study.

### Device assembly

**Fig. 1:**
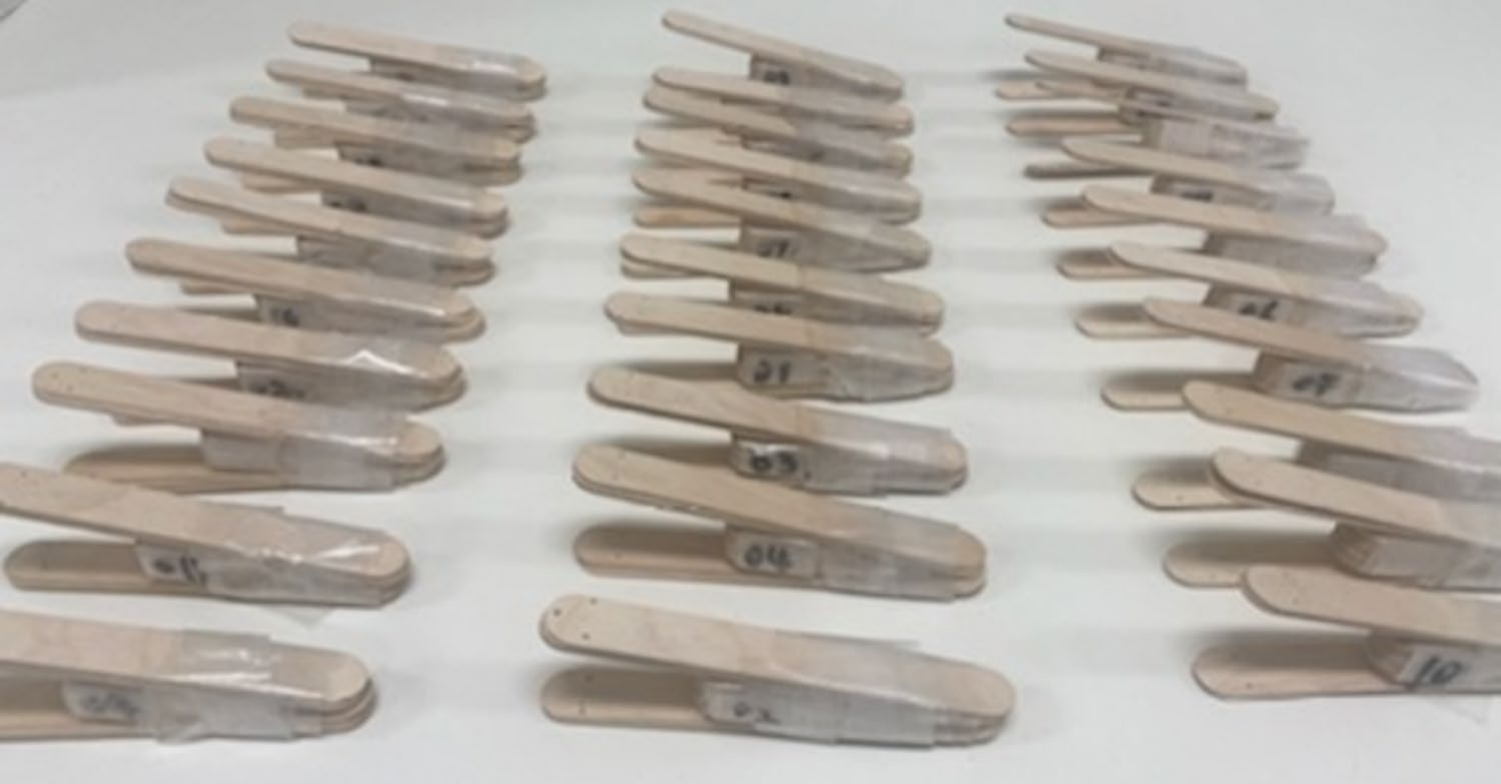
Thirty samples of ARK-JSD, 10 units of easy, medium, and hard levels of resistance

The first author (EC) followed instructions provided by the ARK-J construction guide to make ten ARK-JSD for each level of resistance (easy/medium/hard), giving a total of 30 samples (Fig. 1). A description of the ARK-JSD is available on https://www.youtube.com/watch?v=9NWFCxbJ96Y; however, the construction guide is only available to clinicians who have undertaken the ARK-J course. The device is inserted between the teeth and dentition by narrowing the outer wings; once the device is resting on the dentition or gingiva, the narrowed wings can be released deploying force downwards upon the mandible. Higher resistances are achieved by more tongue depressors added to the design. IID usually refers to the measure between the top and bottom incisors and is measured in millimetres. For the purposes of this study, this number was calculated by measuring from the upper surface of the top tongue depressor to the lower surface of the bottom tongue depressor. Adding resistance increases the IID required to fit the ARK-JSD in the mouth. The ARK-JSD is made to carry out passive (stretch open) and active (bite down) exercises which involve the user to move between minimal IID (mouth biting down and closing the device) to maximal IID (stretching open the device).

### Force and interincisal distance (IID) assessment

**Fig. 2:**
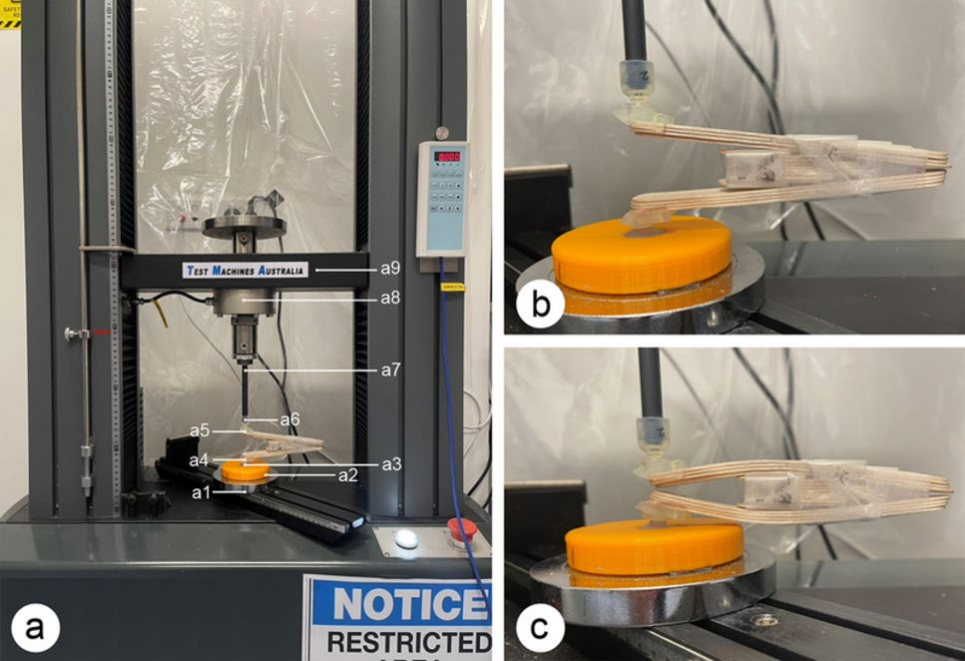
**a** ARK-J Universal Testing Machine setup. (a1) Stationary bottom platen, (a2) 3D printed base disc, (a3) lower ball joint, (a4) lower holding component, (a5) upper holding component, (a6) upper ball joint, (a7) compression upper platen, (a8) load cell, and (a9) movable cross head. **b** Open status of the ARK-J. **c** Close status of the ARK-J

Assessors (KC and AL) were blinded, and force-displacement curves were generated for each of the 30 samples in random order using a TMA-10W Universal Testing Machine (UTM) (Test Machine Australia, Melbourne, Australia) as shown in Fig. 2. A testing jig was designed to prevent the device from slipping whilst under load, and the force was measured corresponding to the interincisal distance (IID) in dentate patients. The jig was 3D printed using a Form3 Stereolithography (SLA) printer and Durable Resin v1 (Formlabs, Somerville, MA, USA), as well as a Raise Pro 2 Fuse Deposition Modelling (FDM) printer and polylactic acid (PLA) filament (Raise3D Technologies, Inc., Irvine, CA 92618, USA). Two frame-holding components were fitted onto the ARK-JSD and connected to two ball joints. The lower ball joint was fitted into a 3D-printed base disc that was fixed to the TMA-10W UTM stationary bottom plate. The compression upper plate, which was coupled to a load cell and movable cross head, was inserted into the upper ball joint’s socket. When the ARK-JSD is loaded, the contacts between the ball joints and the holding components are aligned and remain straight.

### Cyclic fatigue testing

Cyclic testing was performed to assess fatigue over 1750 cycles, replicating a typical exercise regime for trismus therapy of 25 repetitions per day for 30 s, 7 days per week, for 10 weeks, oscillating between minimum and maximum IID; these values represent the full range of each device. Cyclic fatigue testing was performed using the same jig and the UTM setup for three ARK-JSDs (one from each level of resistance).

### Statistical analysis

Statistical analysis was carried out with system software R i386 3.2.2 (The R Foundation for Statistical Computing). Clinically meaningful variation in force was defined as > 5 N, taken as ± 2.5 N from the mean force generated for each ARK-JSD level of resistance. This value was selected based on the experience of the authors using a purpose-built trismus device (Restorabite™) which does have known regulated and incremental force values, where a difference in force of 5 N could consistently be detected by patients. The linearity of force IID curves was assessed using Pearson’s correlation coefficient (*ρ*). High linearity was defined as *ρ* > 0.7, moderate linearity was defined as *ρ* between 0.50 and 0.7, and poor linearity if *ρ* < 0.5 [10]. Force comparisons were standardised according to the minimum and maximal IIDs.

## Results

### IID

**Fig. 3:**
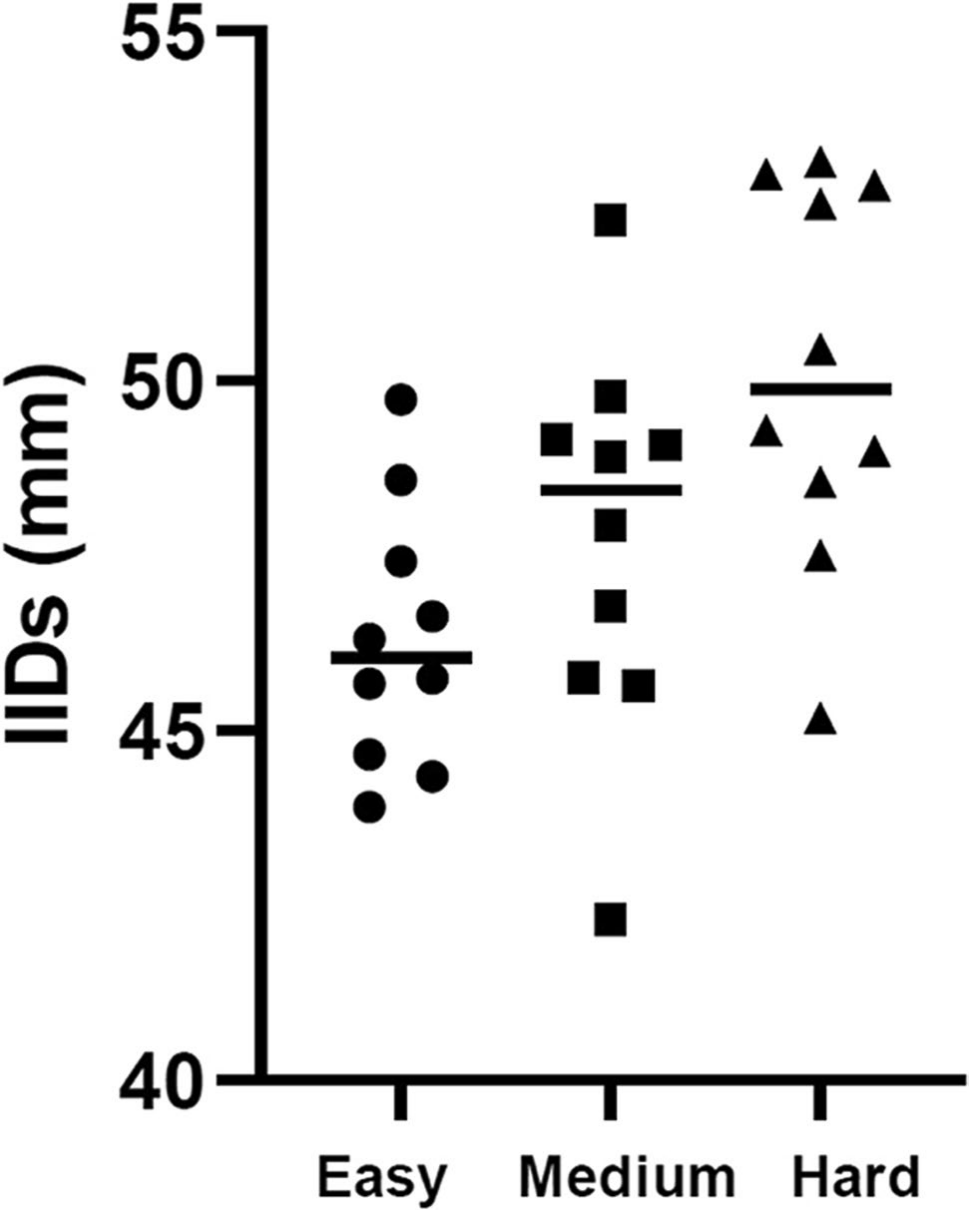
Average maximum IID for easy, medium, and hard ARK-JSDs

The minimum IID for the easy, medium, and hard devices was 8 mm, 13 mm, and 16 mm, respectively. These differences result from the height of the tongue depressors stacked together in the upper and lower part of the frame. The mean maximum IID for the easy, medium, and hard levels of resistance was 46.3 mm (range 43.9–49.7), 47.8 mm (range 42.3–52.3), and 50.1 mm (45.2–53.1) (Fig. 3).

### Force (Table 1)

**Table 1:** Multivariable linear regression models of force for the easy, medium, and hard ARK-JSDs

ARK-JSD level of resistance	Mean (N)	Standard deviation (N)	Range (N)	*p* value	95% CI
Easy	12.3	2.16	9.26–16.1	-	11.0–13.6
Medium	21.0	5.14	15.38–32.94	(Easy vs medium) 0.0003	17.8–24.2
Hard	32.7	6.93	24.68–48.8	(Easy vs hard) < 0.0001	28.4–37.0

*N* Newtons

**Fig. 4:**
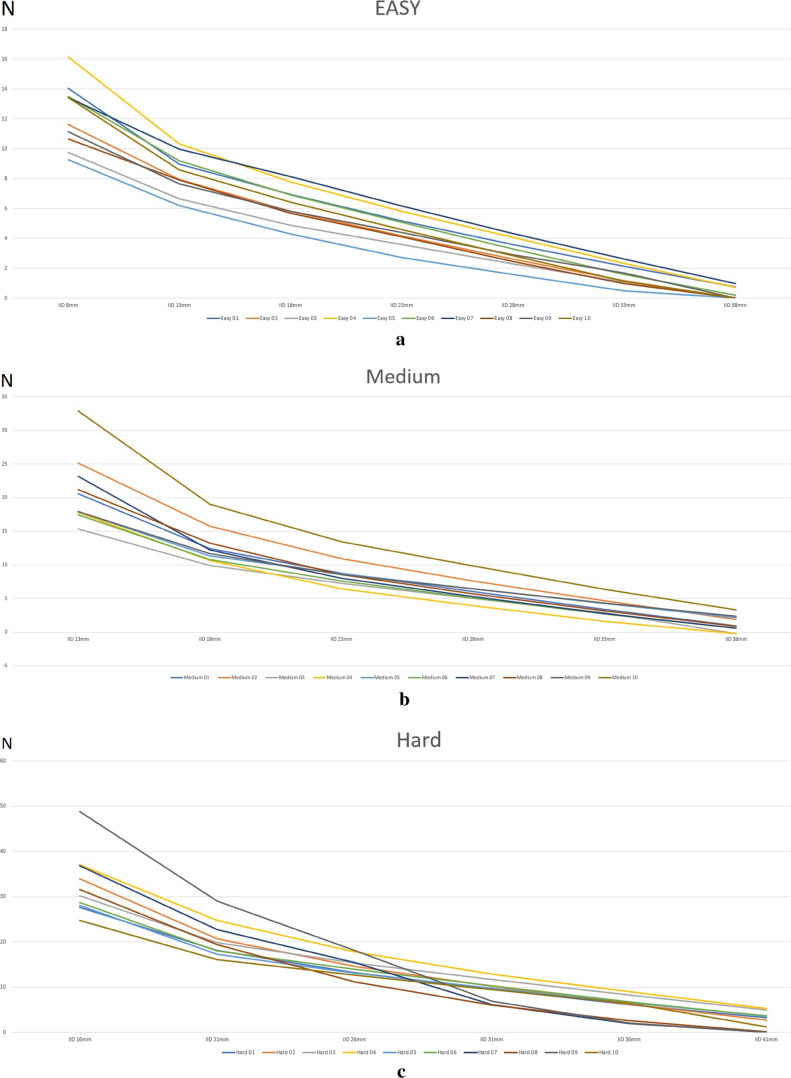
**a** ‘Easy’ force values at specified IID values. **b** ‘Medium’ force values at specified IID values. **c** ‘Hard’ force values at specified IID values

The maximum force generated by the easy ARK-JSD (12.3 ± 2.16 N) was significantly different to the medium ARK-JSD (21.0 ± 5.14 N, *p* = 0.0003), which was significantly different to the mean maximum force generated by the hard ARK-JSD (32.7 ± 6.93 N, p = 0.0005). The maximum force generated by the easy, medium, and hard ARK-JSDs varied by 6.9 N (9.3–16.1 N), 17.6 N (15.4–32.9 N), and 24.1 N (24.7–48.8 N), respectively. When compared to the mean force generated for each level of resistance, this represented a clinically meaningful difference in force (5 N) in 30% of samples of easy, 40% of medium, and 70% of hard ARK-JSDs. The force–displacement curves (Fig. 4) showed poor linearity for the easy ARK-JSD (*ρ* = 49.4 ± 1.8) and moderate linearity for both the medium (*ρ* = 54.2 ± 1.5) and hard ARK-JSDs (*ρ* = 58.6 ± 2.4). They also depict the relationship between IID and force, where in each ARK-JSD, as the IID increased, the force decreased.

### Cyclic fatigue testing

**Fig. 5:**
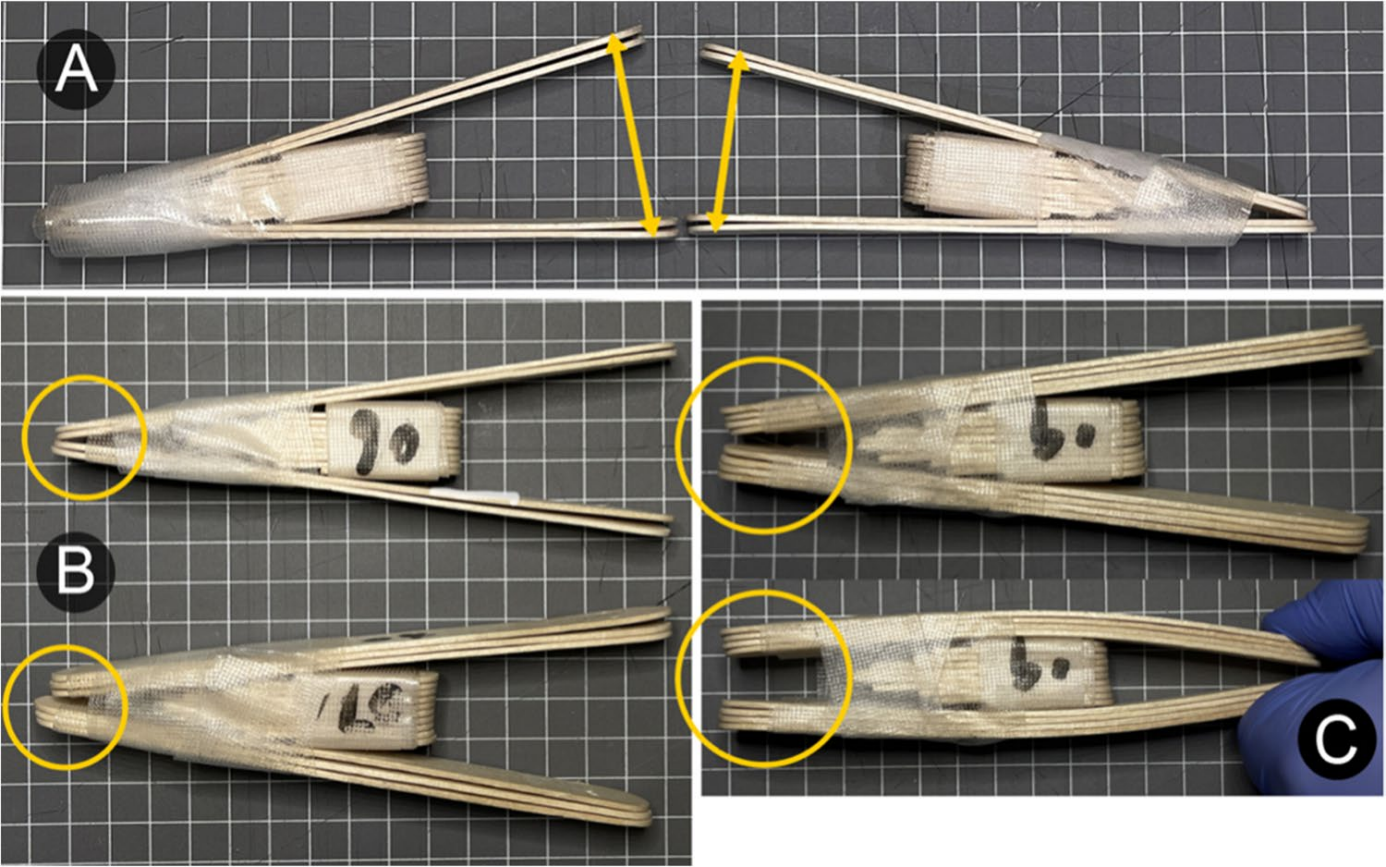
Reasons for loss of force during cyclic testing. **A** Difference in maximal IID for the easy ARK-JSD. **B** Stacked tongue depressor position shifted forward during cyclic testing. **C** Inner wedge shifted forward during cyclic testing

The easy, medium, and hard ARK-JSD experienced a loss in force of 0.4 N (16.1–15.7 N), 3.4 N (25.2–21.8 N), and 5.5 N (48.8–43.3 N), respectively, over accelerated 10 weeks of cyclic testing (1750 cycles of 30 s). The loss of force arose from three factors shown in Fig. 5: (a) changes in maximal IID, (b) misalignment of the outer frame, and (c) loosening of the tape.

## Discussion

This study is the first to describe the objective resistance provided by the ARK-JSD, supporting its use as a low-cost alternative to commercial trismus devices and an evidence-based alternative to stacked tongue depressors. The forces generated by the ARK-JSD all fall below the reported fracture threshold load for a healthy mandible (431–725 N) [11] and below normal bite forces tolerated by healthy dentition (83.9–1642.8 N) [12]. The amount of force exerted was much higher when the ARK-JSD was in the closed position, where the IID was low, and got lower as the IID increased. The force applied by stacked tongue depressors and ARK-JSD is concentrated over a smaller area than TheraBite®; however, the capacity to manipulate the position within the mouth is a distinct advantage of both of these rehabilitation options. Whilst the ARK-JSD is safe to use in a healthy population, the mandibular fracture and dental damage threshold for those recovering from head and neck cancer treatment are unknown and may be considerably lower. We have found that there are several key device reliability factors for clinicians to be aware of. The first is that there is clinically meaningful variation in the maximal force provided for each level of resistance. This may impact the amount of stretch that a patient is able to achieve and impair rehabilitation when swapping between devices, either due to device failure or when progressing between different levels of resistance. The second is the loss of force over time, with the hard ARK-JSD losing over 5.5 N. This is important given that stretching and strengthening generally require force to increase over time for clinical gains.

The ARK-JSD is an affordable trismus device that offers passive and active stretching exercises at three different levels of resistance. It was created to address some of the major barriers to accessing commercial trismus devices such as cost. Whilst existing literature supports the use of commercial trismus devices (e.g. TheraBite®) for people recovering from head and neck cancer [8], access to best-practice care is not always affordable or available. The ARK-JSD design is similar to the Engstrom device. The Engstrom device has been evaluated in comparison to TheraBite® and found to improve mouth opening by a mean IID increase of 6.4 mm [13] and 6.2 mm [14] over a 10-week period. Whilst ARK-JSD has not been evaluated in a prospective trial, the added benefit of three resistance levels that allows for the progression of exercise difficulty is promising. Its low cost and ease of construction using readily available materials make it accessible to patients regardless of socio-economic status, a critical factor lacking in most commercially available trismus devices.

Safety is a primary concern for medical devices, and there is a paucity of data regarding the force that jaw-stretching devices are capable of exerting. This is particularly important in devices, such as the ARK-JSD that rely on a resistance mechanism that is not controlled by the user. Hence, it is the responsibility of the prescribing clinician to understand the maximal force that the device can potentially deliver. Variables such as treatment modalities, age, smoking status, medications, and bone density will lower the dental or jaw fracture threshold, and, in most cases, the ‘safe’ force that can be applied to a jaw following head and neck cancer is unknown [15]. There are currently no simple methods to determine the safe force threshold based on individual patient factors. Instead, trismus therapy replicates guidelines from static stretching literature where the patient stops the intensification of stretches when (or ideally prior to) experiencing pain. In patients recovering from head and neck cancer treatment where sensation is often compromised, the pain signal for force overload may be impaired or absent thus increasing the risk of fracture. Whilst finite element modelling (FEM) based on individual computerized tomography (CT) data could be used to quantify fracture thresholds, this technology is not clinically available, validated, nor practicable.

The ARK-JSD is not designed to, nor would it be expected to meet the durability of commercially made devices. However, whilst the construction protocol was followed for assembling each ARK-JSD unit, there was substantial variation in the maximal force for each resistance level. The greatest variation was observed in the hard ARK-JSD (24.7–48.8 N) which overlapped with the medium frame. Construction variables include how tight a clinician wraps the tape around the device, the presence of any splintering of the tongue depressors in the process of construction, and the position of the wedge within the outer frame (Fig. 5). This variation may affect the ability of patients with trismus to continue to improve their IID over time and should be factored into any rehabilitation plan or if this device was subject to a clinical trial. Despite the variation, there were statistically significant and clinically meaningful differences in the resistance provided by the easy, medium, and hard ARK-JSDs, with sufficient within-level consistency to be effective in most clinical scenarios (Table 1).

### Limitations

There were several limitations inherent in this study including potential inconsistencies in device assembly and limited sample size, and that cyclic testing was not performed on all devices. Furthermore, there is no data regarding what constitutes a clinically meaningful difference in force for trismus therapy. The authors selected 5 N based on their clinical experience; however, this value requires validation. Determining the minimum amount of force change that is discernible in trismus and healthy populations would serve to improve the validity of these findings.

## Conclusion

This is the first study to the author’s knowledge that comprehensively evaluates the force applied to the jaw from a mechanically loaded device. The findings have implications for both treatment efficacy and safety. The ARK-JSD is a novel, low-cost device which features three differing levels of resistance averaging 12.3 N, 21.0 N, and 32.72 N, respectively. Within each level, there is a substantial force variation, and cyclic fatigue testing demonstrates a reduction of up to 5.5 N over 10 weeks. Clinicians should be aware of not only its limitations but also the opportunities offered by this device.

## Data Availability

The datasets generated and/or analysed during the current study are available from the corresponding author on reasonable request.
